# Women’s Attitudes Toward Fertility and Childbearing: A National Cross-Sectional Study in Saudi Arabia

**DOI:** 10.3390/healthcare13202616

**Published:** 2025-10-17

**Authors:** Deemah Ateeq AlAteeq, Ebtihag O. Alenzi, Reema Abdulrahman Alamri, Abeer Abdulkarim Aloraini, Dimah Saif Alassaf, Nujud Ibrahim Almutlaq, Shatha Saleh Aloglla, Albatool Abdullah Almajhad, Rana Hussain Jahhaf

**Affiliations:** 1 Internal Medicine Department, College of Medicine, Princess Nourah bint Abdulrahman University, P.O. Box 84428, Riyadh 11671, Saudi Arabia; 2 Family and Community Medicine Department, College of Medicine, Princess Nourah bint Abdulrahman University, P.O. Box 84428, Riyadh 11671, Saudi Arabia; 3 College of Medicine, Princess Nourah bint Abdulrahman University, P.O. Box 84428, Riyadh 11671, Saudi Arabia

**Keywords:** attitudes, childbearing, fertility, motherhood, women

## Abstract

**Background:** The decline in fertility rates is a growing concern globally, impacting family dynamics and the economy. This study assesses women’s attitudes toward fertility and childbearing in Saudi Arabia and explored their associations with sociodemographic, medical, and psychological factors. **Methods:** This one-year cross-sectional study involved 2172 Arabic-speaking women aged 18–50 years in Saudi Arabia, with the data collected between December 2022 and January 2023. The online survey, distributed through posts on social media apps as WhatsApp, Twitter, Telegram, Snapchat, and Facebook, measured sociodemographic characteristics, medical and psychiatric history, childbearing preference, and the Arabic version of the Attitudes toward Fertility and Childbearing Scale (AFCS). **Results:** Item–total correlations ranged from r = 0.124 to higher, all significant at *p* < 0.01, supporting the scale’s validity. Exploratory Factor Analysis (EFA) identified four emerging factors (eigenvalues > 1), and Bartlett’s test confirmed sample suitability (*p* < 0.001; Kaiser–Meyer–Olkin = 0.898). Cronbach’s alpha coefficient was 0.898. Participants who were young (71.5%), unmarried (72%), and had psychiatric disorders (16.5%) were more likely to perceive childbearing as less important for their future. Nevertheless, these same groups, along with students (61%), frequently regarded childbearing as a present hindrance that necessitates preparation. Furthermore, participants who were undecided about having children (22.5%) exhibited lower scores in the female identity domain. **Conclusions:** The Arabic version of the Attitudes toward Fertility and Childbearing Scale appears to be psychometrically sound, encompassing four factors: importance for the future, hindrance at present, childbearing preparation, and female identity. This study found that younger women and those with psychiatric disorders showed lower scores regarding the importance of future childbearing, while college students exhibited more concerns about hindrance and preparation.

## 1. Introduction

Over the past years, the marked decrease in the fertility rate has been acknowledged as one of the most prominent global changes [1]. Fertility rate indicates the number of live births per 1000 women of reproductive age in a specific year [2]. Various factors influence it, such as economic conditions, social norms, healthcare access, and personal attitudes toward childbearing [3]. In line with these global influences, Saudi Arabia has experienced a steep decline in fertility indices [4]. The World Bank estimates that Saudi Arabia’s birth rate has decreased alarmingly from 44 births per thousand inhabitants in 1980 to 17 births per thousand in 2020, and the global ranking has reached 102 according to the World Development Indicators (WDI) classification [5]. Currently, Saudi Arabia’s total fertility rate stands at approximately two children per woman, which is below replacement level [5].

This decline highlights the importance of understanding women’s intentions and attitudes toward childbearing, as these are critical factors that influence family planning and could further shape fertility trends and family structure [6]. To better examine these influences, it is essential to distinguish between fertility intentions, which are defined as explicit plans or decisions regarding having children, and attitudes toward childbearing, which refer to personal feelings or perceptions about having children, whether positive or negative [7]. These distinctions are especially relevant, as many young women tend to postpone childbearing for various reasons [8]. Fertility intentions can be further categorized into forward-looking and retrospective types [9]. While forward-looking intentions focus on future plans to have children, retrospective intentions reflect past experiences or circumstances related to childbearing. Both types are shaped by factors such as social expectations, career goals, and financial stability [9].

Given the growing tendency among young women to delay childbearing and the complex factors shaping fertility intentions and attitudes, sustaining this decline in fertility rates could bring about major issues, particularly economic challenges such as a reduced workforce, which may hinder investments and consumption [10]. Furthermore, this shift will impose significant burdens, resulting in a widening generational gap and notable changes in family structures [11]. A reduction in fertility rates often leads to a higher proportion of elderly people in society, which presents challenges for social services and healthcare systems [12]. The drop in birth rates is a well-known reality, and it is essential to recognize and address this shift [3]. Certainly, this will impact the progression of growth initiatives, as sustainable development programs rely on the equilibrium between increasing income levels and population density [12].

In the context of fertility attitudes, positive attitudes toward having children can be influenced by factors such as family support, stable relationships, and career stability. Research in the United States revealed that positive attitudes toward childbearing were associated with earlier parenthood, especially among married women [13]. Building on the importance of understanding these attitudes, a Swedish study developed the Attitudes Toward Fertility and Childbearing Scale (AFCS) as a tool for systematically assessing individuals’ perspectives on childbearing [14]. This study found that students rated the importance of future fertility higher than unemployed women. Additionally, women living in larger cities were more likely to see childbearing as a hindrance [14]. Another Swedish study found that having a supportive partner and being sufficiently mature were the most important elements for women’s decision to have children [15]. Also, another study conducted among Danish female healthcare professionals demonstrated that most women hoped to have children and valued parenting, and half of them underestimated the impact of age on female fertility [16]. In addition, a recent Korean study showed that university students wanted to have children, but the ability to balance work and family life, along with access to childcare options, impacted this desire [17]. Furthermore, another study has demonstrated a relationship between psychological disorders and attitudes toward having children [18]. Collectively, these findings underscore the importance of differentiating between fertility intentions, defined as specific plans or decisions about whether and when to have children, and attitudes toward childbearing, which reflect an individual’s emotional or cognitive responses to the idea of having children, ranging from highly positive to highly negative [10]. While fertility intentions are widely recognized as strong predictors of reproductive behaviors and their outcomes (child or no child), previous research has shown that there is limited understanding of the reasons behind fertility intentions [19]. Therefore, this research focuses on women’s attitudes toward childbearing rather than their future intentions. To our knowledge, there is a noticeable gap in research exploring women’s attitudes toward childbearing in Saudi Arabia. Although a national study among female health professions students found that most of them expressed a desire to have children, most of the respondents intended to postpone starting a family until they have a secure job [20].

Understanding women’s attitudes toward fertility and childbearing has direct clinical implications. These attitudes affect reproductive decision-making, contraceptive use, and the timing of pregnancies, all of which influence maternal and child health outcomes. In clinical practice, being aware of these perspectives allows for healthcare providers to offer more personalized counseling, identify women at risk of delayed or unplanned pregnancies, and address mental health or psychosocial factors that may impact reproductive choices. In Saudi Arabia, where cultural norms and rapid socioeconomic changes intersect, assessing these attitudes is crucial for integrating reproductive health services with mental health support and ensuring that women’s health needs are comprehensively met. Understanding women’s attitudes toward fertility and childbearing is not only clinically relevant for tailoring reproductive counseling and integrating mental health screening, but is also vital for shaping public health policy. These insights enable policymakers to design culturally sensitive, targeted interventions, particularly for younger women, students, and those with psychiatric disorders, while ensuring that reproductive health services, educational initiatives, and supportive workplace or academic policies align with national family planning and population health goals.

To assess whether different groups have distinct views on fertility, we employed known-groups validity by analyzing the attitudes of women across multiple categories, including age, marital status, education, occupation, region of residence, and other relevant factors. We predicted significant differences in attitudes based on these attributes. Specifically, younger women were expected to exhibit more idealistic views on fertility, while older women would demonstrate more realistic views. Married women were hypothesized to hold more positive attitudes toward childbearing than their unmarried counterparts. Further, we predicted that educational level and occupation would be associated with attitudes, with postgraduate women and employed women showing more deliberate and self-determined views on family planning [5].

The purpose of this study is to assess women’s attitudes toward fertility and childbearing in Saudi Arabia and to explore how sociodemographic, medical, and psychological factors are associated with these attitudes. The primary objective is to assess the attitudes of Saudi women toward childbearing, while the secondary objectives include examining the role of sociodemographic factors (e.g., age, education, employment), medical history, and psychological factors in shaping these attitudes.

Research questions:

What are the attitudes of Saudi women toward childbearing?

Are sociodemographic factors such as age, education, and employment associated with Saudi women’s attitudes toward fertility and childbearing?

What role do medical and psychological factors play in shaping women’s attitudes toward having children in Saudi Arabia?

## 2. Methods

### 2.1. Study Design

This one-year cross-sectional descriptive observational study was carried out among Arabic-speaking women aged 18–50 in Saudi Arabia, whose data were collected between December 2022 and January 2023, adhering to the principles outlined in the Helsinki Declaration. Ethical clearance was granted by the Institutional Review Board (IRB) of Princess Nourah bint Abdulrahman University, Riyadh, Saudi Arabia (IRB Log Number: 22-1093) on 15 November 2022. Voluntary participation was required, informed consent was obtained from all participants, and full clarification of the purpose of the study was provided to the participants prior to data collection. The study ensured confidentiality and data protection by anonymizing all responses and storing data securely.

### 2.2. Sample

The study consisted of two phases: a pilot study and the main study. The pilot study phase enrolled 250 Arabic-speaking women and was conducted to assess the psychometric properties (validity and reliability) of the translated Arabic version of the Attitudes to Fertility and Childbearing Scale (AFCS). It was originally created by Stherberg et al., using the Swedish language. In previous studies, this tool has been translated and validated using different languages in different countries [14,21,22,23]. However, there has yet to be a validated Arabic version of this scale. Therefore, the pilot study was essential to ensure that the Arabic version of the AFCS was suitable for use in the Arabic population before proceeding with the main study.

Using G-power software (version 3.1.9.6), the minimum required sample size was calculated to be 970, based on a 95% power, a 95% confidence level, and a margin of error of ±5%. This was adjusted to 1261 to compensate for an anticipated 30% non-response rate. After the validation of the AFCS through the pilot study, a sample of 2172 women in Saudi Arabia was collected using Google Forms, a secure web-based survey administration software. Snowball and convenience sampling techniques were employed. The study investigators distributed the survey link, with inclusion criteria of being a member of the Saudi general population aged 18–50, through social media apps such as WhatsApp, Twitter, Telegram, Snapchat, and Facebook. Those younger than 18 or older than 50 years, non-Saudis, and those with incomplete questionnaires were excluded.

### 2.3. Data Collection

The questionnaire was distributed in Arabic and consisted of 43 questions divided into four sections:

(a) Sociodemographic characteristics: age, current residency, marital status, education level, occupation status, and income.
(b) Medical and psychiatric history: diagnosis of medical conditions, diagnosis of psychiatric conditions, infertility, and contraceptive methods used.
(c) Childbearing preference: sex and timing of having children.
(d) Attitudes toward Fertility and Childbearing Scale (AFCS): This scale evaluates attitudes toward fertility and childbearing. It consists of 27 items (see Table 3), each rated on a 5-point Likert scale ranging from 1 to 5 (5 = highly agree, and 1 = highly disagree). The original and first version of AFCS measure the construct (women’s attitudes to fertility and childbearing) with the main three factors: (1) Importance of Fertility for the Future with nine items (1 to 9); (2) Childbearing as a Hindrance at Present with 12 items (10 to 21); and (3) Social Identity with six items (22 to 27). It was used in previous research in many languages [21,22,23,24]. The Arabic version of the AFCS was developed following standard forward–backward translation procedures. Two independent bilingual experts—one with a background in reproductive health and the other in psychology independently translated the original English scale into Arabic. A third bilingual expert, blinded to the original scale, performed the back-translation into English. Discrepancies were discussed and resolved by a committee consisting of translators and two senior researchers to ensure conceptual equivalence, cultural appropriateness, and clarity. The pre-final version was then evaluated for content validity by a panel of five subject-matter experts (two reproductive health specialists, one psychiatrist, one sociologist, and one public health researcher). Each expert independently rated the relevance and clarity of each item using Davis’s method, calculating both item-level (I-CVI) and scale-level (S-CVI) Content Validity Index scores. Items with I-CVI values below 0.80 were revised accordingly. The final Arabic version of the AFCS was then pilot-tested on 250 Arabic-speaking women to confirm comprehension and assess its validity and reliability before its use in the main study. It demonstrated very good to excellent validity and reliability. Cronbach’s alpha coefficients ranged from 0.833 to 0.934. The exploratory factor analysis (EFA) showed four emerging factors (eigenvalues > 1) using the Oblimin rotation method. The instrument has four factors, which were labeled as follows: (1) Importance of Fertility for the future with nine items (F1 to F9), (2) Childbearing as a Hindrance at Present with nine items (H1, H2, H4, H5, H6, H7, H8, H9, H10), (3) Childbearing preparation with six items (H3, H11, H12, S3, S4, S5) and (4) Female identity with three items (S1, S2, S6). Following the successful validation in the pilot phase, the main study was conducted using he larger sample (*n* = 2,172) to address the primary research questions regarding Saudi women’s attitude toward fertility and childbearing and their associations with sociodemographic, medical, and psychological factors.

### 2.4. Statistical Analysis

Statistical analysis was performed with SPSS Version 25 and JASP 0.95. Descriptive statistics are shown as means with standard deviations (SDs) for continuous variables, and as frequencies with percentages for categorical variables. The validity of the AFCS items was tested using the Pearson product-moment correlation method, with a *p*-value (≤0.05) and the critical value table of the Pearson Product-Moment Correlation Coefficient (r > 0.124) [24]. Construct validity was examined through exploratory factor analysis (EFA) for structure detection, with principal component analysis (PCA) employed for item reduction to determine the emerging factors in the Arabic version. The Oblimin rotation method was used for expected correlated factors. The sufficiency and suitability of the sample for running factor analysis using Bartlett’s test of sphericity, which is suggested to have a significant *p*-value (less than 0.05), and the Kaiser-Meyer-Olkin Measure of Sampling Adequacy (KMO), which is supposed to be loaded above 0.6 [25,26]. The number of factors was decided based on Kaiser’s criterion of having eigenvalues higher than one [27]. Items were retained if they had a primary pattern coefficient loading of ≥0.40 and a cross-loading of at least 0.20 across factors. Items showing cross-loading but demonstrating strong conceptual alignment with their respective factors and contributing positively to internal consistency were also retained.

Reliability (internal consistency) was evaluated for the whole scale and each emerging component using Cronbach’s alpha coefficients and McDonald’s omega coefficients with their 95% confidence intervals (95% CI) to provide a more robust estimate of internal consistency. The coefficients are required to be ≥0.70 to be acceptable [28]. The correlational model of new factors was plotted, and significant correlations (Pearson correlation) were presented. Both independent *t*-tests and one-way ANOVA tests were used to verify the statistical significance among different variables. *p*-values less than 0.05 were considered significant.

## 3. Results

### 3.1. Pilot Study Results: Psychometric Validation of the Arabic AFCS (n = 250)

#### 3.1.1. The Sociodemographic Characteristics and State of Health

The psychometric properties of the Arabic version of the AFCS were assessed using the pilot sample (*n* = 250).

#### 3.1.2. Reliability

Table 1 shows very good estimates of internal consistency, with a Cronbach’s alpha coefficient of 0.0898. Also, the findings showed that the scale score reliability of the items in the four factors ranged from good to excellent, as indicated by Cronbach’s alpha coefficients with values ranging from 0.833 to 0.934. Likewise, the scale and its factors perform very well in McDonald’s Omega, as all scores were higher than 0.70, which indicates excellent reliability.

#### 3.1.3. Validity

The validity of all items of the total scale using the Pearson correlation was presented in Table 2. The result of the intercorrelation between the items and the total score of the AFCS showed that the item “S4” had the highest score (r = 0.702) and the item “H10” had the lowest score (r = 0.327). All items had r values significantly higher than the Pearson Product-Moment Correlation Coefficient’s critical value table (r > 0.124, *p* < 0.01).

**Figure 1:**
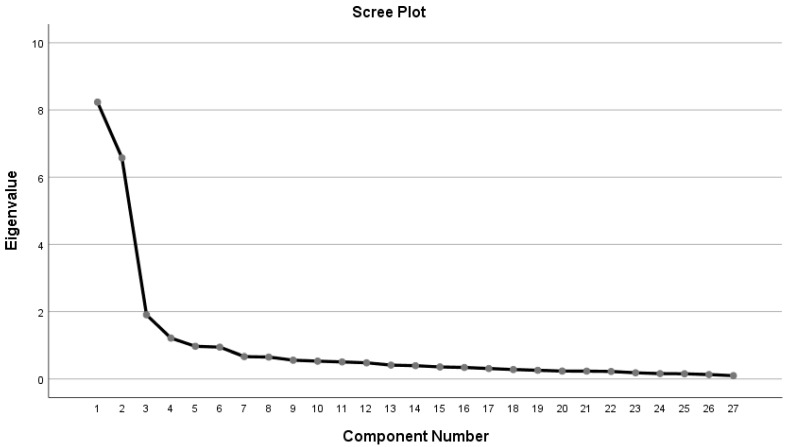
Scree plot for items of factors that emerged from the Arabic version of the AFCS.

The EFA construct validity results revealed four emerging factors (eigenvalues > 1), as demonstrated in Figure 1. Parallel analysis confirmed that the first four observed eigenvalues exceeded those generated from random data, supporting the retention of four factors. This finding was consistent with the inflection point observed in the scree plot, further justifying the four-factor solution. The factor loadings with the structure and pattern coefficients are shown in Table 3. With a cumulative percentage of variation of 70.10%, the extraction sum of the squared loadings is 18.94. Results from Bartlett’s test of sphericity were significant, with a *p*-value less than 0.001, and the KMO was 0.898, which loaded above 0.6. These findings indicated that the sample is sufficient and suitable for running factor analysis. Although most items showed clear primary factor loadings (≥0.40), a few demonstrated moderate cross-loadings. For example, item H4 was retained under “Childbearing as a Hindrance at Present” due to its strong conceptual relevance and its positive contribution to the factor, despite loading above 0.40 on more than one factor.

The scree plot shows that four factors had eigenvalues greater than 1, indicating the emergence of four factors in the EFA. The curve levels off after the fourth factor, suggesting minimal additional variance explained by subsequent factors.

**Figure 2:**
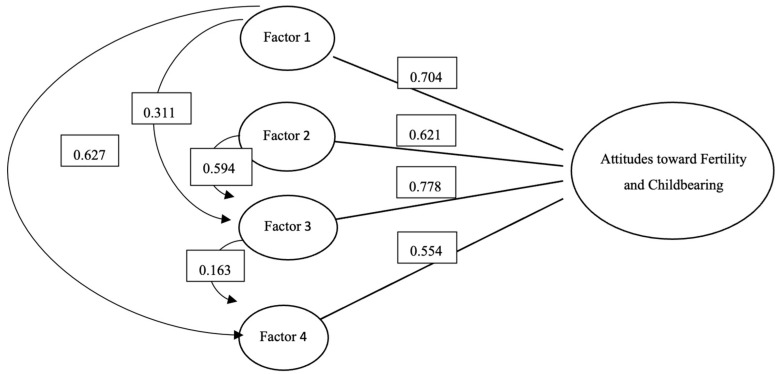
The four-factor correlational model of the Arabic version of the AFCS.

Figure 2 shows the correlational model of the four factors in the Arabic version of the AFCS. All factors were significantly correlated with the AFCS.

### 3.2. Main Study Results: Attitudes Toward Fertility and Childbearing (n = 2172)

Following validation of the AFCS in the pilot phase, the main study was conducted using a large sample of 2172 Arabic women to explore their attitudes towards fertility and childbearing, and their associations with various sociodemographic, medical, and psychological variables.

The sociodemographic characteristics, medical and psychiatric history, and childbearing preference of the sample are presented in Table 4. Most of them aged 18–25 years (71.5%), were unmarried (72%), without kids (77.6%), grew up with parents (91.1%), were students (61%), and had a bachelor’s degree (78%). More than half of them have enough family income (52.2%). Participants were from the five regions of Saudi Arabia; however, 40.8% lived in the Central region. Also, 12.1% and 16.5% of them were diagnosed with medical and psychiatric conditions. Only 3.8% were pregnant, 10.7% used contraceptives, and 3% had infertility problems. Moreover, more than a third of participants prefer to have kids after the first two years of marriage (34.3%), and 62.6% of the participants had not decided what sex they prefer for their future child.

Table 5 presents the average scores for each item and the average score for the total AFCS, calculated as a summation of all items (79.14 ± 19.42). For the items, item (H10) achieved the lowest result (2.31 ± 1.06), while item (S5) achieved the highest result (3.85 ± 1.47).

Table 6 displays the women’s attitudes toward fertility and childbearing and their relationship with the study’s variables.

Overall, the analysis identified several significant factors associated with women’s attitudes toward fertility, including age, marital status, polygamy, education, occupation, region of residence, having children, and the timing of the decision to have children.

(1) Importance for the future

The mean score for women’s attitudes toward the importance of childbearing in their future was 26.20 ± 9.44. Significant factors associated with women’s attitudes toward the importance of future childbearing included: age, marital status, parental status (having children), pregnancy status, occupation, psychiatric history, region of residence, and decision regarding having children. A lower score in this domain was associated with being aged 18–25 (25.54 ± 9.08, *p* < 0.001), unmarried (25.23 ± 8.87, *p* < 0.001), not having kids (25.53 ± 9.02, *p* < 0.001), not being pregnant (26.07 ± 9.38, *p* < 0.001), being a student (25.42 ± 9.10, *p* < 0.001), having a psychiatric disorder (24.76 ± 9.51, *p* < 0.002), and not making decision to have a child (22.63 ± 8.17, *p* < 0.001).

(2) Hindrance at present

The mean score for hindrance to childbearing at present was (24.40 ± 8.50). Significant factors associated with hindrance scores included: age, marital status, residence region, having children, pregnancy status, occupation, psychiatric history, decision about having children, and preference for the child’s sex. Significantly higher hindrance scores were observed among women aged 18–25-year (25.66 ± 8.66, *p* < 0.001), unmarried (25.71 ± 8.58, *p* < 0.001), residing in the central region (25.52 ± 8.82, *p* < 0.001), not having kids (25.35 ± 8.53, *p* < 0.001), not being pregnant (24.53 ± 8.52, *p* < 0.001), being a student (25.92 ± 8.82, *p* < 0.001), having a psychiatric disorder (26.38 ± 9.19, *p* < 0.001), did not decide of having kids (26.19 ± 9.02, *p* < 0.001), and having a preferable sex (25.01 ± 8.42, *p* < 0.009). In contrast, lower hindrance scores were found among women residing in the northern region (22.42 ± 8.07, *p* < 0.001), who reported using contraceptives (21.93 ± 7.49, *p* < 0.001), having a medical condition (23.13 ± 8.93, *p* < 0.01), and experiencing infertility problems (20.86 ± 8.60, *p* < 0.001).

(3) Childbearing preparation

The mean score of women’s attitudes toward preparation for childbearing was 18.21 ± 5.21. Significantly higher scores were observed among women who aged 18–25-year (18.53 ± 5.08, *p* < 0.001), unmarried (18.46 ± 5.08, *p* < 0.001), not having kids (18.42 ± 5.08 *p* < 0.001), being a student (18.55 ± 5.15, *p* = 0.001), having a psychiatric disorder (18.77 ± 5.30 *p* = 0.027), deciding to have children after the first two years of marriage (18.75 ± 5.05, *p* < 0.001), and having a preferred sex (18.66 ± 5.08 *p* = 0.002).

(4) Female identity

The mean score of female identity in childbearing was 10.33 ± 3.54. Significantly lower scores were observed among women who did not decide to have a child (9.36 ± 3.32, *p* < 0.001) and those who did not have a preferred sex for their child (10.20 ± 3.59, *p* = 0.031).

## 4. Discussion

This study is regarded as the first in Saudi Arabia to evaluate women’s attitudes towards fertility and childbearing, as well as the first to examine their relationships with various factors, utilizing a valid and reliable Arabic version of the AFCS.

Although the AFCS has been used widely among countries with different languages, such as Polish, Turkish, Persian, and Japanese, there was no version for the Arabic women population [15,23,29]. Thus, the AFCS was translated and tested using a sample of Arabic women of reproductive age. The Arabic version of the AFCS demonstrates high internal consistency (Cronbach’s alpha = 0.898 and McDonald’s omega = 0.853) and significant item intercorrelations with the average score of total AFCS (r > 0.124, *p* < 0.01), confirming the scale’s validity and reliability. Factor analysis identified four key factors explaining 70.10% of the variance, supported by Bartlett’s test of sphericity (*p* < 0.001) and a KMO value > 0.6. Among the items, the one about whether having children would limit socializing had the lowest average score (2.68) and the lowest intercorrelation with the total AFCS score (r = 0.327), indicating minimal impact on social life. Conversely, the item about the importance of having a stable relationship when having children scored the highest (4.30). In contrast, the item with the highest intercorrelation (r = 0.702) was one’s life must be prepared for living with children. Unlike previous studies, such as the Swedish study, which identified three factors Importance of fertility for the future,” “Childbearing as a hindrance at present,” and “Social identity.” [14], factor analysis in this study revealed four factors. The “importance for future” factor had a strong positive correlation with female identity and a moderate positive correlation with childbearing preparation. In contrast, childbearing preparation had a weak positive correlation with female identity, but strongly correlated with hindrance at present. These results affirm the scale’s effectiveness and applicability for Arabic-speaking women. 

This study reveals that most participants valued the importance of fertility and childbearing for their future, particularly those who were married, had children, preferred to have children within the first year of marriage, and were aged 36–49 years. This finding aligns with previous studies emphasizing the cultural and social importance of marriage and parenthood in Saudi Arabia [20]. Similarly, another study has shown that many women plan to have children during periods of significantly reduced fertility, increasing the risk of unintended childlessness or challenges in conceiving later in life [15].

In interpreting the finding that women who expressed a preference for the sex of their future children were more committed to having children, it is important to consider the broader sociocultural context. In our sample, these attitudes may reflect deeply held cultural norms, familial expectations, and identity-related values that frame childbearing as both a personal and social milestone. While this relationship may appear self-evident, our results suggest that it is reinforced by normative beliefs around motherhood and gender roles, particularly in settings where family formation is strongly valued. Likewise, the concept of “hindrance” warrants unpacking, as its meaning may differ across groups. For some participants, hindrance referred to financial constraints such as the cost of childcare or housing; for others, it encompassed limited family or social support, including the absence of extended family help; and for yet others, it involved career-related interruptions and concerns about professional advancement. Recognizing these varied dimensions highlights that perceived barriers to childbearing are not uniform, but are instead shaped by intersecting economic, social, and occupational factors.

Additionally, this study reveals that having a psychiatric disorder may negatively impact women’s perception of the significance of future fertility, as they prefer to be prepared for childbearing while viewing it as a current hindrance. This indicates that psychological factors influence women’s decision-making about childbearing in Saudi Arabia. Living with a psychiatric disorder is a dysfunctional life factor that affects self-care and quality of life, making daily tasks burdensome [24]. Similarly, a previous study found that participants with a family history of psychiatric disorders reported being either completely unwilling or less inclined to have children [18].

In contrast to the Swedish study, the present study found that female students anticipated a lower score on the importance of fertility for their future compared to unemployed women [14]. The age differences could mediate this association between students who are more likely to be younger and unemployed women who might be older.

This study found that age, marital status, and occupation were significant variables of childbearing. Unmarried participants aged 18–25 and students perceived greater barriers to childbearing and expressed a desire to feel more prepared for it, which resulted in lower scores regarding its future importance. The previous literature suggested that the presence of a supportive partner and financial stability play a significant role in women’s decisions to have children [14,25]. This may explain why younger groups, such as unmarried students, perceive more obstacles [14]. These findings highlight the importance of family planning and education in Saudi Arabia, showcasing a trend where women postpone childbearing until they complete their education and establish their careers. Prior research indicated that 85% would postpone starting a family until completing their education and securing a fulfilling job [14].

Moreover, this study shows that women in the Central and Eastern regions consider childbearing a significant obstacle. This finding is consistent with a previous study that indicated that women in larger cities are more likely to view childbearing as a hindrance compared to those in mid-sized cities [14]. Furthermore, women with a preference for a specific gender rated higher in the child preparation aspect. This suggests that preferring a particular sex for a future child may influence the time they take to prepare.

This study also evaluates female identity, revealing that planning to have children within the first two years of marriage and expressing a preference for the sex of a future child are associated with a stronger sense of female identity. This may reflect the cultural and social expectations of women in Saudi Arabia, where motherhood is often viewed as a defining aspect of female identity and a crucial element of fulfilling gender roles [15]. Consequently, the lower scores among women who have not decided to have children may reflect ambiguity or uncertainty about their roles and perceptions as women within their cultural context [14]. This uncertainty may arise from various factors, including age, educational and career aspirations, and societal pressures. Furthermore, the importance placed on having a preferred sex for future children aligns with the notion that traditional gender expectations can influence attitudes toward childbearing [28]. Preferences of a specific gender for future children might reflect conformity to societal norms and expectations, reinforcing traditional concepts of female identity [29].

Overall, this study offers valuable insights into the attitudes of women in Saudi Arabia regarding fertility and childbearing, highlighting the considerable influence of sociodemographic, medical, and psychological factors. The findings emphasize the cultural and social significance of marriage and parenthood, especially among older, married women with children, while also uncovering the impact of psychiatric disorders on fertility perceptions. The study identifies key variables related to perceived barriers to childbearing, including age, marital status, occupation, and residence, noting that younger, unmarried, and student participants encounter more obstacles. Furthermore, it highlights the role of education and career establishment in preparing for childbearing. Lastly, the study illustrates the connection between early childbearing and a strong sense of female identity, reflecting the traditional gender roles and cultural expectations in Saudi Arabia. These findings can guide public health initiatives and inform women’s health researchers in developing programs that support reproductive health and decision-making in the region.

This study’s strengths include a large, regionally diverse sample, potentially improving its generalizability results, and the study population reflects a broad sociodemographic, medical, and psychological spectrum. Furthermore, this is the first study to evaluate women’s attitudes toward fertility and childbearing in Saudi Arabia using a valid scale.

## 5. Limitations

While this study has notable strengths, limitations must be considered. The use of convenience sampling could introduce selection bias. In addition, as a cross-sectional study, it is not feasible to establish causal relationships between the variables. Given the robust sample size and comprehensive EFA conducted, we believe the current validation provides sufficient and reliable evidence of the Arabic AFCS structure. Nevertheless, future studies could consider employing confirmatory factor analysis (CFA) with independent samples to further strengthen the structural validity of the scale.

## 6. Conclusions

In conclusion, the Arabic version of the AFCS demonstrated strong validity and reliability. Key factors influencing women’s attitudes toward fertility and childbearing included younger age, university enrollment, marital status, and the presence of a psychiatric disorder. To support informed family planning decisions, women would benefit from access to reliable information and educational programs about the importance of fertility and childbearing.

Further research is needed to study which psychiatric illnesses could affect women’s attitudes toward childbearing. Additionally, qualitative and longitudinal studies are recommended to track changes in attitude over time. Moreover, while this study focused on women—since the AFCS was developed for females—men’s perspectives on fertility and childbearing are important for further study. Exploring men’s parenting intentions would lead to a more comprehensive understanding of reproductive decision-making in Saudi families.

## Figures and Tables

**Table 1:** Reliability of each factor and the whole scale, and the intraclass correlation coefficient (ICC).

Items	Cronbach’s Alpha [95% CI]	r (ICC)	McDonald’s Omega [95% CI]	*p*-Value
All AFCS items	0.898 [0.867–0.929]	0.882 excellent	0.853 [0.827–0.879]	<0.01 *
Factor 1 (F1–F9)	0.934 [0.919–0.949]	0.925 excellent	0.935 [0.922–0.947]	<0.01 *
Factor 2 (H1, H2, H4–H10)	0.881 [0.856–0.906]	0.870 excellent	0.886 [0.865–0.907]	<0.01 *
Factor 3 (H3, H11, H12, S3–S5)	0.875 [0.836–0.914]	0.851 excellent	0.870 [0.844–0.895]	<0.01 *
Factor 4 (S1, S2, S6)	0.833 [0.787–0.879]	0.832 excellent	0.852 [0.822–0.882]	<0.01 *

ICC: Intraclass Correlation Coefficient. 95% CI: 95% Confidence Interval. * Significant at *p* value lower than 0.05.

**Table 2:** Pearson correlation between each item and the total score.

Questions	*r*	*p*-Value
(F1) I look forward to one day becoming a mother	0.561	<0.01 *
(F2) Having children is an essential part of life	0.557	<0.01 *
(F3) Having children will develop me as a person	0.608	<0.01 *
(F4) find it hard to imagine living a life without children	0.427	<0.01 *
(F5) I can imagine being pregnant and giving birth	0.577	<0.01 *
(F6) Having a child is a way for me to add new elements in life	0.595	<0.01 *
(F7) I talk to my friends about having children in the future	0.484	<0.01 *
(F8) It is important for me to be fertile	0.696	<0.01 *
(F9) It is important for me to be able to get pregnant anytime	0.663	<0.01 *
(H1) Having children would limit my life right now	0.475	<0.01 *
(H2) An unplanned pregnancy would hinder me in my current life	0.449	<0.01 *
(H3) Childbearing does not fit into my life right now	0.443	<0.01 *
(H4) Taking responsibility for a child does not fit into my current life	0.421	<0.01 *
(H5) Having children would limit my leisure time activities	0.500	<0.01 *
(H6) I do not want to take responsibility as a mother now	0.409	<0.01 *
(H7) Having children would limit my career	0.431	<0.01 *
(H8) Being a mother would take too much of my own time	0.536	<0.01 *
(H9) Having children would limit my study opportunities	0.462	<0.01 *
(H10) Having children would limit socializing with my friends	0.327	<0.01 *
(H11) It is important for me to choose when to get pregnant	0.603	<0.01 *
(H12) It is important for me to have my own stable economy when I have children	0.599	<0.01 *
(S1) My fertility makes me feel communion with other women	0.449	<0.01 *
(S2) Being fertile is important for my identity as a woman	0.497	<0.01 *
(S3) It is important to me that the child is born in a nuclear family, i.e., mother, father, children	0.688	<0.01 *
(S4) When I have children, my life must be prepared for living with children	0.702	<0.01 *
(S5) It is important for me to have a stable relationship when I have children	0.699	<0.01 *
(S6) Becoming a mother is important for my identity as a woman	0.492	<0.01 *

Pearson correlation. * Significant at *p* value lower than 0.05.

**Table 3:** Factor loadings and the AFCS items’ score coefficients (pattern and structure matrix) with a four-factor solution (*n* = 250).

Items	Factor Number *							
	1 (Importance for Future)		2 (Hindrance at Present)		3 (Childbearing Preparation)		4 (Female Identity)	
	Pattern Coefficients	Structure Coefficients	Pattern Coefficients	Structure Coefficients	Pattern Coefficients	Structure Coefficients	Pattern Coefficients	Structure Coefficients
(F1) I look forward to one day becoming a mother	**0.831**	**0.844**	−0.017	−0.069	−0.078	−0.202	0.000	0.357
(F2) Having children is an essential part of life	**0.785**	**0.840**	−0.017	−0.078	−0.034	−0.163	0.114	0.446
(F3) Having children will develop me as a person	**0.643**	**0.710**	−0.025	0.022	−0.280	−0.376	0.048	0.349
(F4) find it hard to imagine living a life without children	**0.753**	**0.784**	0.004	−0.130	0.168	0.033	0.137	0.435
(F5) I can imagine being pregnant and giving birth	**0.778**	**0.801**	0.036	−0.008	−0.082	−0.222	0.032	0.369
(F6) Having a child is a way for me to add new elements in life	**0.669**	**0.802**	−0.056	0.069	−0.111	−0.224	0.261	0.556
(F7) I talk to my friends about having children in the future	**0.716**	**0.753**	0.017	−0.065	0.039	−0.091	0.105	0.402
(F8) It is important for me to be fertile	**0.750**	**0.811**	0.099	0.103	−0.192	−0.359	0.096	0.437
(F9) It is important for me to be able to get pregnant anytime	**0.768**	**0.788**	0.146	0.127	−0.144	−0.324	0.028	0.373
(H1) Having children would limit my life right now	0.277	0.245	**0.448**	**0.438**	−0.046	−0.256	0.014	0.153
(H2) An unplanned pregnancy would hinder me in my current life	−0.092	−0.126	**0.476**	**0.630**	−0.386	−0.543	−0.118	−0.093
(H4) Taking responsibility for a child does not fit into my current life	−0.426	−0.314	**0.416**	**0.642**	−0.454	−0.571	0.196	0.086
(H5) Having children would limit my leisure time activities	−0.062	−0.081	**0.592**	**0.704**	−0.275	−0.494	−0.008	0.021
(H6) I do not want to take responsibility as a mother now	−0.386	−0.340	**0.550**	**0.737**	−0.370	−0.535	0.102	0.004
(H7) Having children would limit my career	−0.060	−0.131	**0.815**	**0.811**	0.029	−0.282	0.034	0.039
(H8) Being a mother would take too much of my own time	0.063	−0.016	**0.640 **	**0.741**	−0.292	−0.534	−0.148	−0.062
(H9) Having children would limit my study opportunities	0.004	−0.072	**0.861 **	**0.831**	0.080	−0.260	0.053	0.081
(H10) Having children would limit socializing with my friends	0.088	0.110	**0.847**	**0.727**	0.287	−0.055	−0.011	0.028
(H11) It is important for me to choose when to get pregnant	0.111	0.110	0.0.287	0.519	**−0.645**	**−0.753**	−0.174	−0.042
(H12) It is important for me to have my own stable economy when I have children	−0.015	0.141	−0.029	0.301	**−0.840**	**−0.833**	0.053	0.141
(H3) Childbearing does not fit into my life right now	−0.364	−0.256	0.0.361	0.605	**−0.523**	**−0.620**	0.126	0.047
(S3) It is important to me that the child is born in a nuclear family, i.e., mother, father, children	0.227	0.388	−0.082	0.209	**−0.800**	**−0.811**	0.065	0.248
(S4) When I have children, my life must be prepared for living with children	0.234	0.401	−0.093	0.206	**−0.819**	**−0.828**	0.072	0.259
(S5) It is important for me to have a stable relationship when I have children	0.227	0.391	−0.047	0.243	**−0.822**	**−0.842**	−0.046	0.162
(S1) My fertility makes me feel communion with other women	−0.034	0.324	0.028	0.077	−0.027	−0.129	**0.849**	**0.839**
(S2) Being fertile is important for my identity as a woman	0.186	0.523	−0.012	−0.013	0.040	−0.077	**0.814**	**0.887**
(S6) Becoming a mother is important for my identity as a woman	0.381	0.641	−0.057	−0.087	0.04	−0.064	**0.622**	**0.775**

* Significant at *p* value lower than 0.05.

**Table 4:** Sample characteristics and childbearing preference (*n* = 2172).

Variables	*n*	%
**Age (years)**		
18–25	1552	71.5%
26–35	372	17.1%
36–49	248	11.4%
**Marital status**		
Non-married	1568	72%
Currently Married	604	28%
**Polygamy**		
First wife	688	31.7%
Second, third, fourth wife	45	2.1%
Unmarried	1439	66.2%
**Education**		
Postgrad degree	141	6%
Bachelor’s degree	1691	78%
High school or less	340	16%
**Occupation**		
Student	1328	61%
Employed	309	14%
Unemployed	535	25%
**Residence**		
Western region	692	31.9%
Central region	886	40.8%
Northern region	142	6.5%
Eastern region	278	12.8%
Southern region.	174	8%
**Kids**		
No	1685	77.6%
Yes	487	22.4%
**Pregnant**		
No	2090	96.2%
Yes	82	3.8%
**Living with parents**		
No	194	8.9%
Yes	1978	91.1%
**Income**		
Enough	1133	52.2%
Enough with saving	535	24.6%
Not enough	354	16.3%
In debt	150	6.9%
**Contraceptives**		
No	1939	89.3%
Yes	233	10.7%
**Medical condition**		
No	1910	87.9%
Yes	262	12.1%
**Infertility problem**		
No	2107	97.0%
Yes	65	3.0%
**Self-reported Psychiatric disorder**		
No	1813	83.5%
Yes	359	16.5%
**Decision to have kids**		
First year of marriage	206	9.5%
After the first year of marriage	733	33.7%
After the first 2 years of marriage	744	34.3%
Not decided	489	22.5%
**Preferable sex**		
No	1359	62.6%
Yes	813	37.4%

**Table 5:** AFCS total and item means (*n* = 2172).

Items	Mean ± SD
(F1) I look forward to one day becoming a mother	3.15 ± 1.46 [1–5]
(F2) Having children is an essential part of life	3.11 ± 1.44 [1–5]
(F3) Having children will develop me as a person	2.99 ± 1.38 [1–5]
(F4) I find it hard to imagine living a life without children	2.60 ± 1.25 [1–5]
(F5) I can imagine being pregnant and giving birth	2.83 ± 1.35 [1–5]
(F6) Having a child is a way for me to add new elements in life	2.91 ± 1.38 [1–5]
(F7) I talk to my friends about having children in the future	2.49 ± 1.24 [1–5]
(F8) It is important for me to be fertile	3.13 ± 1.44 [1–5]
(F9) It is important for me to be able to get pregnant anytime	2.99 ± 1.39 [1–5]
(H1) Having children would limit my life right now	2.59 ± 1.15 [1–5]
(H2) An unplanned pregnancy would hinder me in my current life	2.69 ± 1.27 [1–5]
(H3) Childbearing does not fit into my life right now	2.85 ± 1.43 [1–5]
(H4) Taking responsibility for a child does not fit into my current life	2.80 ± 1.41 [1–5]
(H5) Having children would limit my leisure time activities	2.78 ± 1.30 [1–5]
(H6) I do not want to take responsibility as a mother now	2.72 ± 1.41 [1–5]
(H7) Having children would limit my [1–5]	2.55 ± 1.19 [1–5]
(H8) Being a mother would take too much of my own time	2.88 ± 1.37 [1–5]
(H9) Having children would limit my study opportunities	2.55 ± 1.19 [1–5]
(H10) Having children would limit socializing with my friends	2.31 ± 1.06 [1–5]
(H11) It is important for me to choose when to get pregnant	3.26 ± 1.45 [1–5]
(H12) It is important for me to have my own stable economy when I have children	3.66 ± 1.49 [1–5]
(S1) My fertility makes me feel communion with other women	2.60 ± 1.16 [1–5]
(S2) Being fertile is important for my identity as a woman	2.62 ± 1.28 [1–5]
(S3) It is important to me that the child is born in a nuclear family, i.e., mother, father, children	3.76 ± 1.47 [1–5]
(S4) When I have children, my life must be prepared for living with children	3.72 ± 1.48 [1–5]
(S5) It is important for me to have a stable relationship when I have children	3.85 ± 1.47 [1–5]
(S6) Becoming a mother is important for my identity as a woman	2.76 ± 1.36 [1–5]
AFCS TOTAL	79.14 ± 19.4 [27–135]

Descriptive test (mean and standard deviation); SD: Standard deviation.

**Table 6:** Women’s attitudes toward fertility and childbearing among different groups.

Attributes		Importance for Future		Hindrance at Present		Childbearing Preparation		Female Identity	
		x̄ ± SD	*p*-Value (Effect Size)	x̄ ± SD	*p*-Value (Effect Size)	x̄ ± SD	*p*-Value (Effect Size)	x̄ ± SD	*p*-Value (Effect Size)
Age (Years)	18–25	25.54± 9.08 ^≠¥^	*p* < 0.001 (0.01) **	25.66 ± 8.66 ^≠¥^	*p* < 0.001 (0.05) **	18.53 ± 5.08 ^≠¥^	*p* < 0.001 (0.02) **	10.37 ± 3.41 ^≠¥^	0.500 (0.01) **
	26–35	27.72 ± 9.97 ^β^		22.12 ± 7.16 ^β^		17. 87± 5.56 ^β^		10.31 ± 3.70 ^β^	
	36–49	28.04 ± 10.35 ^β^		19.92 ± 7.01 ^β^		16.75 ± 5.24 ^β^		10.09 ± 4.02 ^β^	
Marital status	Unmarried	25.23 ± 8.87	*p* < 0.001 (0.36) *	25.71 ± 8.58	*p* < 0.001 (0.59) *	18.46 ± 5.08	*p* < 0.001 (0.17) *	10.33 ± 3.40	0.969 (0.00) *
	Married	28.73 ± 10.38		20.99 ± 7.25		17.55 ± 5.50		10.33 ± 3.86	
Polygamy	First wife	27.99 ± 10.38 ^¥^	*p* < 0.001 (0.02) **	21.30 ± 7.41 ^¥^	*p* < 0.001 (0.06) **	17.62 ± 5.67 ^¥^	*p* < 0.001 (0.02) **	10.27 ± 3.87 ^¥^	0.173 (0.01) **
	Second, third, fourth wife	26.31 ± 9.90		20.60 ± 7.56		16.27 ± 5.35		9.42 ± 4.03	
	Unmarried	25.34 ± 8.83		26.00 ± 8.57		18.56 ± 4.94		10.33 ± 3.35	
education	Postgrad degree	26.20 ± 9.80	0.454 (0.000) **	22.09 ± 6.84 ^≠^	0.003 (0.004) **	17.67 ± 5.46	0.305 (0.003) **	10.04 ± 3.66	0.242 (0.000) **
	Bachelor Degree	26.32 ± 9.49		24.62 ± 8.55 ^β^		18.29 ± 5.15		10.40 ± 3.47	
	High school or less	25.61 ± 9.06		24.23 ± 8.72		18.02 ± 5.39		10.11 ± 3.77	
Occupation	Student	25.42 ± 9.10 ^≠¥^	*p* < 0.001 (0.01) **	25.92 ± 8.82 ^≠¥^	*p* < 0.001 (0.04) **	18.55 ± 5.15 ^≠¥^	0.001 (0.01) **	10.35 ± 3.40 ^≠¥^	0.948 (0.01) **
	Employed	28.02 ± 10.36 ^β^		21.79 ± 7.63 ^β^		17.76 ± 5.59 ^β^		10.28 ± 3.77 ^β^	
	Unemployed	27.07 ± 9.53 ^β^		22.12 ± 7.21 ^β^		17.64 ± 5.08 ^β^		10.32± 3.72 ^β^	
Residence	Western region	26.50 ± 9.32 ^α^	*p* < 0.001 (0.01) **	23.66 ± 8.16 ^α^	*p* < 0.001 (0.01) **	18.19 ± 5.25 ^α^	0.001 (0.01) **	10.47 ± 3.59 ^α^	0.001 (0.01) **
	Central region	26.62 ± 9.49 ^α^		25.52 ± 8.82 ^α^		18.61 ± 4.90 ^α^		10.47 ± 3.36 ^α^	
	Northern region	25.82 ± 9.76 ^α^		22.42 ± 8.07 ^α^		17.01 ± 6.08 ^α^		9.37 ± 3.87 ^α^	
	Eastern region	26.56 ± 9.43 ^α^		24.32 ± 7.84 ^α^		18.27 ± 5.15 ^α^		10.45 ± 3.56 ^α^	
	Southern region	22.59 ± 8.74		23.36 ± 8.82		17.15 ± 5.66		9.67 ± 3.70	
Kids	No	25.53 ± 9.02	*p* < 0.001 (0.31) *	25.35± 8.53	*p* < 0.001 (0.53) *	18.42 ± 5.08	*p* < 0.001 (0.18) *	10.35 ± 3.42	0.615 (0.02) *
	Yes	28.53 ± 10.47		21.09 ± 7.51		17.47 ± 5.60		10.26 ± 3.91	
Pregnant	No	26.07 ± 9.38	*p* < 0.001 (0.01) *	24.53 ± 8.52	*p* < 0.001 (0.03) *	18.22 ± 5.20	0.600 (0.12) *	10.33 ± 3.53	0.823 (0.02) *
	Yes	29.57 ± 10.41		21.15 ± 7.26		17.91 ± 5.54		10.24± 3.69	
Living with parents	No	26.29 ± 10.04	0.890 (0.01) *	23.95± 8.90	0.440 (0.02) *	18.21 ± 5.52	0.998 (0.00) *	10.45 ± 3.66	0.609 (0.01) *
	Yes	26.19 ± 9.39		24.44 ± 8.46		18.21 ± 5.18		10.32 ± 3.52	
Income	Enough	26.27 ± 9.46	0.235 (0.001) **	24.37 ± 8.54	0.843 (0.000) **	18.20 ± 5.24	0.867 (0.000) **	10.40± 3.57	0.646 (0.000) **
	Enough with saving	26.68 ± 9.49		24.62 ± 8.53		18.36 ± 5.01		10.23 ± 3.38	
	Not Enough	25.40 ± 9.20		24.34 ± 8.52		18.09 ± 5.42		10.19 ± 3.61	
	In debt	25.80 ± 9.64		23.93 ± 8.08		18.07 ± 5.23		10.47 ± 3.64	
Contraceptives	No	26.13 ± 9.37	0.316 (0.01) *	24.69 ± 8.56	*p* < 0.001 (0.12) *	18.28 ± 5.18	0.070 (0.05) *	10.36 ± 3.50	0.329 (0.01) *
	Yes	26.79 ± 10.02		21.93 ± 7.49		17.63 ± 5.45		10.12 ± 3.79	
Medical condition	No	26.21 ± 9.31	0.834 (0.01) *	24.57 ± 8.42	0.010 (0.03) *	18.25 ± 5.14	0.293 (0.08) *	10.37 ± 3.49	0.184 (0.01) *
	Yes	26.08 ± 10.36		23.13 ± 8.93		17.89 ± 5.71		10.06 ± 3.85	
Infertility problems	No	26.18 ± 9.38	0.522 (0.03) *	24.51 ± 8.47	0.001 (0.23) *	18.24 ± 5.19	0.113 (0.12) *	10.34 ± 3.52	0.263 (0.03) *
	Yes	26.94 ± 11.38		20.86 ± 8.60		17.20 ± 5.74		9.85 ± 3.97	
Psychiatric Disorder	No	26.48 ± 9.41	0.002 (0.13) *	24.01 ± 8.30	*p* < 0.001 (0.12) *	18.10 ± 5.19	0.027 (0.05) *	10.35 ± 3.57	0.489 (0.01) *
	Yes	24.76 ± 9.51		26.38 ± 9.19		18.77 ± 5.30		10.21 ± 3.37	
Decision of Having kids	First year of marriage	29.15 ± 10.98 ^¥^	*p* < 0.001 (0.01) **	19.84 ± 7.90 ^≠¥^	*p* < 0.001 (0.01) **	17.32 ± 5.98 ^≠¥^	*p* < 0.001 (0.02) **	10.09 ± 4.04 ^≠¥^	*p* < 0.001 (0.001) *
	After the first year of marriage	28.77 ± 9.69		22.46 ± 7.20		18.40 ± 5.22		10.92 ± 3.63	
	After first 2 year of marriage	25.20 ± 8.53		26.40 ± 8.59		18.75 ± 5.05		10.45 ± 3.28	
	Not decided	22.63 ± 8.17		26.19 ± 9.02		17.48 ± 4.98		9.36 ± 3.32	
Preferable sex	No	25.86 ± 9.42	0.30 (0.05) *	24.03 ± 8.53	0.009 (0.03) *	17.94 ± 5.27	0.002 (0.02) *	10.20 ± 3.59	0.031 (0.001) *
	Yes	26.77 ± 9.46		25.01 ± 8.42		18.66 ± 5.08		10.54 ± 3.43	
Total		26.20 ± 9.44		24.40 ± 8.50		18.21 ± 5.21		10.33 ± 3.54	

ANOVA and *t*-test was used. Post hoc analysis: ^β^ Significant difference with first variable. ^≠^ Significant difference with second variable. ^¥^ Significant difference with third variable. ^α^ Significant difference with fifth variable. * Cohen’s d to assess effect size for *t*-test analysis (small effect: d ≈ 0.2, medium effect d≈0.5, large effect d ≈ 0.8). ** Eta-squared to assess effect size in ANOVA analysis (small effect Eta-squared ≈ 0.01, medium effect Eta-squared ≈ 0.06, and large effect Eta-squared ≈ 0.14).

## Data Availability

Data are available upon request due to privacy restrictions.
